# Solvent for Salicylic Acid

**Published:** 1877-02

**Authors:** D. W. Edmiston

**Affiliations:** Clinton, Ill.


### SOLVENT FOR SALICYLIC ACID.

CLINTON, ILL., December, 1876.

Allow me to call your attention to the solvent alcohol for salicylic acid. The proportions are as follows :

Salicylic Acid, . . . . . . . . . . . . . gr. 30.

Alcohol 95 per cent. . . . . . . . . gr. 49½ Bulk ℥ iss.

Mix. Will form a complete solution without chemical change in the above proportions. Add distilled water, gr. 60 Bulk ℥ i. It will re-crystalize the entire quantity in solution into an anhydrous mass. Weight, gr. 134—dry, 24 gr. It will readily dry on filtering paper exposed to the air, leaving the residue a beautiful needle crystal, very fine, but well defined under a high power. Other agents containing alcohol will make a solution for all practical purposes, as Spts. Lavender, Spts. Rosemary, etc.

Aqueous solutions must be studiously avoided as incompatible, while ammoniated agents are admissible with morphine and an alchoholic menstrum of opium.

Yours, D. W. Edmiston, M.D.

